# Human major transitions from the perspective of distributed adaptations

**DOI:** 10.1098/rstb.2021.0401

**Published:** 2023-03-13

**Authors:** Ehud Lamm, Meir Finkel, Oren Kolodny

**Affiliations:** ^1^ The Cohn Institute for History and Philosophy of Science and Ideas, Tel Aviv University, Tel Aviv 69978, Israel; ^2^ Department of Ecology, Evolution, and Behavior, Institute for Life Sciences, The Hebrew University of Jerusalem, Jerusalem 9190401, Israel

**Keywords:** collective memory, extended mind, group selection, niche construction, collective action, collective decision making

## Abstract

Distributed adaptations are cases in which adaptation is dependent on the population as a whole: the adaptation is conferred by a structural or compositional aspect of the population; the adaptively relevant information cannot be reduced to information possessed by a single individual. Possible examples of human-distributed adaptations are song lines, traditions, trail systems, game drive lanes and systems of water collection and irrigation. Here we discuss the possible role of distributed adaptations in human cultural macro-evolution. Several kinds of human-distributed adaptations are presented, and their evolutionary implications are highlighted. In particular, we discuss the implications of population size, density and bottlenecks on the distributed adaptations that a population may possess and how they in turn would affect the population's resilience to ecological change. We discuss the implications that distributed adaptations may have for human collective action and the possibility that they played a role in colonization of new areas and niches, in seasonal migration, and in setting constraints for minimal inter-population connectivity.

This article is part of the theme issue ‘Human socio-cultural evolution in light of evolutionary transitions’.

## 1. Human evolutionary transitions involve changes in distribution of knowledge

Major transitions in evolution involve the emergence of new units of selection, changes in levels of organization and complexity, and—respectively—new ways in which adaptive information, in various forms, is stored and used. This paper is concerned with the origins and implications of new ways of representing and storing adaptive information in the course of human evolution. Specifically, we focus on the often-hidden assumption that adaptively relevant information is at the level of the individual, in the sense of being stored by the individual in genes, in the brain, or even in the composition of its microbiome, and that it can be transmitted from one individual to another.

By contrast, we focus on the recently proposed concept of *distributed adaptations* (DAs) [1], cases in which adaptive information strictly transcends the level of individuals, and we explore the role of this kind of adaptation in human evolution. We will use the terms information and knowledge interchangeably to refer to that which confers adaptive value. It seems that few would argue against the observation that human culture transcends the knowledge and abilities of individuals and that adaptive benefits often arise from combining information from multiple individuals. This truism may, however, be cashed out in different ways which imply different evolutionary considerations (see also [2] and related commentaries, as well as [3,4]). We will offer a precise notion of cultural knowledge that captures the intuition that this knowledge is greater than the sum of individual knowledge. Most theoretical models of cultural evolution assume that information is acquired through social learning, by individuals, and track information that is, strictly speaking, at the level of individuals. The results, of course, may manifest at the population level, and may be shaped by cultural group selection [5]. A particularly central case is cumulative culture [6–10]. It is typically assumed that cumulative culture in humans is decomposable to individual-level pieces of information, found in a population. Here we will start from the assumption that humans use knowledge that is distributed in the population and that might not be readily decomposable to several discrete kinds of knowledge, as would be the case in division of labour or in the skill pool effect [11–13]. Rather than defend this assumption, which we think is fairly non-controversial, we will elucidate evolutionary implications that arise from it, and assess whether these implications of distributed knowledge may be part of the explanation of events in human cultural macro-evolution. A more precise definition of DA and its links to related concepts is found in [1].

Whether adaptive information is distributed is related to, but distinct from, questions concerning units of selection. The philosopher Elisabeth Lloyd articulated four questions to clarify the notions of evolutionary units by distinguishing between what she calls the interactor question, the replicator question, the beneficiary question, and the manifestor-of-adaptation question [14]. Unlike the ordinary cases of simple, individual-level selection on organisms, a population is the level that ‘manifests’ a DA, while individual members of the population may be the beneficiaries. The notion of DA is deliberately agnostic about the processes that allow the DA to replicate or persist, which may involve a complex of genetic, epigenetic, behavioural and symbolic–cultural processes, with feedbacks and redundancy. The units of interaction are likewise complex in the case of DAs because they can involve parts of the physical environment, organisms of other species, and more or less ephemeral mutual interactions among organism-interactors. We mostly focus on cases in which the evolutionary beneficiaries are individuals in the population, that is on cases of multi-level selection 1 [15]. However, the main advantage of the DA perspective is to move beyond discussion of units of selection and allow us to identify interesting ways in which human populations achieve adaptive results, thereby highlighting interesting evolutionary trajectories and constraints [1].

A related line of research focuses on a population's ability to create collective results that go beyond what individuals can achieve, that is, on collective action [8]. The notions of collective action, collective intelligence and collective knowledge, as discussed in the literature, do not presuppose the complexity of the groups and apply to a wide range of cases studied in cultural evolution. Attempts to create bridges linking cultural evolution and collective action have recently been made [2,10]. The theoretical approach we outline in this paper is directly related to such initiatives. Distributed knowledge has the potential to mitigate the costs of collective action problems and promote social coordination. It lowers the cognitive costs of learning and remembering, as well as opening up potential adaptations that are only available at the population levels. However, because distributed knowledge is fundamentally collective, understanding its evolutionary stability requires addressing the usual evolutionary problems that collective action entails.

The notion of DA makes it possible to expose various evolutionary scenarios and processes, in particular those that involve changes in the way information is stored: whether individually or in a distributed fashion; the degree of distribution of information (e.g. the number of copies, etc.); and the coevolution of traits that allow information to be distributed (e.g. acquisition, signalling, data consolidation) and traits whose development or use rely on the distributed information, perhaps the most interesting case.

## 2. Distributed adaptations and human culture

DAs in the strict sense are cases in which adaptation is conferred by some structural or compositional aspect of the population; the adaptively relevant information cannot be reduced to information possessed by a single individual (i.e. in genes or in the brain of an individual). Flocking and herding behaviours arguably fall under this definition, as are uses of stigmergy in social insects, and collective foraging, e.g. in vultures [1]. While there are many theoretical niceties, a population can potentially collect, store and interact about more information or knowledge than is simply the sum of the knowledge of individuals. The affordances of DA, however, support information that is different in quality, not merely quantity. For example, they allow the population to aggregate information from several locations, to retain adaptive information across generations, and more. We discuss examples that we find particularly striking below. The affordances that distributed information offer make DAs candidate drivers of evolutionary transition, since the change in the way information can be used and propagated are due to the shift to a population level. Importantly, when distributed knowledge affects the adaptation of individuals and populations to their environment, selection may favour traits that allow their bearers to make effective use of the distributed knowledge in various ways. The extent of reliance on DAs is itself also shaped by natural selection; selection may favour lineages that become more reliant on such external knowledge or less reliant on it, influenced by factors such as extent of dependence on population size and structure or susceptibility to cheating. An important related question is whether selection can favour lineages or groups that can store a greater amount of distributed information; in other words, whether selection can favour populations that are ‘better’ repositories of distributed knowledge.

Are some cultural adaptations in humans DAs? In simple cases it is possible for an individual to learn what they need to know from a single other individual. Cases of division of labour and of the associated knowledge are clearly DAs, but arguably they are somewhat degenerate cases, as the substantive knowledge (e.g. technical or ecological knowledge) is readily partitioned into parts that can be transmitted from individual to individual. That being said, however, as one of the reviewers of this paper suggested to us, it is plausible that in order to learn from another individual one first need to know/identify that individual's particular role in the group, perhaps also know the cues from other group roles that render credit to this individual's knowledge, and know which of these cues to use and which to ignore. This may indeed be the case in humans in some contexts, specifically in division of labour, and maybe in other species. This is ultimately an empirical question.

A population may embody additional information, such as the frequencies of different behaviours and their payoffs, which organisms may evolve to utilize. In more complex cases, an individual acquires the information it requires by combining information from multiple other individuals (often in a certain order), or individuals engage in a joint endeavour such as brainstorming in which adaptive outcomes are an emergent property of the dynamics. The availability and salience of information may even affect the developmental trajectory of individuals. We discuss below several examples which suggest that humans have used a variety of kinds of DAs.

We will use as a working definition a loose characterization of DAs: cases in which adaptively relevant information that is used by individuals is the result of collective behaviour over time and cannot be transmitted in full from a single individual to another, and in which the distribution of information, in its broad sense, is part of the explanation of the adaptive value of the information. We will also discuss cases in which adaptively relevant information is materially embodied externally of individuals, and cases in which the DA temporally transcends the actions or lifespans of individuals.

Cultural evolutionary theorists have for many years drawn attention to cases that fall under this characterization [2,5,16]. We add to the attempts to study these phenomena by focusing on different types of DAs found in human evolution, the various possible routes by which they can emerge, and finally on the evolutionary implications they have, in particular constraints on population size and population connectivity. Our discussion shows that the existence of DA is orthogonal to the issues of group selection and cultural group selection since DAs potentially arise in other ways. Cultural niche construction is another related notion, but our discussion points to DAs that do not amount to a change to a physical or cultural niche unless the latter term is understood very broadly so as to accommodate the ideas we develop. We return to the differences below. The notion of DA, thus, suggests new avenues for research on phenomena that have long interested researchers on cultural evolution and points to additional related phenomena.

Various phenomena seem to arguably fall under this characterization. One example can be seen in game drive lanes, also called bison jumps or desert kites in different parts of the world [17–22]. These are constructed fences, or walls/lines of rock cairns designed to funnel big game, when being chased, towards a kill site in which they fall off a cliff or are easily targeted in some other way. These represent DA at multiple levels: first, both the construction and the use of these structures required collective action of many individuals. Second, the knowledge regarding how and where to construct such structures, which may span several miles and include thousands of rock piles, and how to operate them, would likely have required the expertise of multiple experienced individuals. Finally, some of these ancient structures were used over thousands of years, thus embodying, and maintaining over time, knowledge that may be of crucial importance, and that was acquired gradually over time, through experience, and that was possibly never possessed by a single individual. These structures can be viewed as adaptations distributed not only across the population but across a multitude of populations that had existed previously and shaped the lanes’ forms.

A second example, sharing many qualitative similarities with game drive lanes, are systems of water collection and irrigation. Diverse water systems are found across the globe, some dating from thousands of years ago, and many that have been in continuous or intermittent use for hundreds of years [23–28]. Such systems often embody adaptive knowledge that might not be known to any single individual during much of the period of the system's use. This may include, for example, where to carve rock-hewn cisterns that would not leak or at which location to dig a well that reaches a highly localized aquifer, as well as complex knowledge that may be an emergent property of years of trial-and-error rather than explicit knowledge that can be verbally conveyed. Such knowledge can relate to the regularity of water flows, to seasonal water availability, and more, and may be embodied in the system's structure or in the societal rules regarding its use [27].

A third example of DAs is the social system itself and elements within it, such as the social institutions of a society and other traditions. These may capture crucially important adaptive knowledge which is often implicit in the institutions’ structure, goals or *modus operandi*. Examples range from simple behavioural rules that may be dictated by social norms, including religious stipulations like the requirement for ritual hand washing before any eating of bread in Judaism (a requirement dating nearly 2000 years prior to the discovery of the existence of bacteria), to the overall structuring of society in a way that reflects adaptation to local conditions and resources, such as division to clans or chiefdoms, the split to professional guilds, the existence of formal social roles such as medicine-people or chiefs, or the physical spread across the landscape which utilizes its resources efficiently. Particularly interesting examples of the latter adaptation are cases in which populations migrate seasonally or split and re-convene annually according to a certain traditional regularity whose ecological reasoning may be opaque to the individuals that practise the behaviour.

Other systems of note, which will be discussed in some detail below, include trail systems, song lines that describe space and routes, as existed traditionally among Aboriginal communities in Australia, and remains—intentional or not—of sites of tool production, which were used repeatedly across eons.

## 3. Distributed adaptations come in different flavours

Clearly, each of these phenomena is different. The perspective of DA allows us to expose theoretically relevant similarities among them, but it is helpful to begin by classifying them roughly into four kinds of DAs employed by humans (i.e. kinds of distributed adaptive information):

1. *Ephemeral DAs*—These are cases in which the information resides in activities or signals and disappears once they stop. During a coordinated activity such as hunting in coordinated groups or in driving game across a large expanse, for example, crucial information for the endeavour's success is captured in the spatial location of the participants and the signalling—intentional or unintentional—among them.
2. *Stable material DAs*—These cases involve ecological changes that are stable for a meaningful period of time beyond that of the particular individuals or even groups that created them. Examples in non-humans include aspects of termite mounds and animal trails (e.g. goat trails). Possible cases of important human stable DAs are: trails and roads; irrigation systems; drive lanes; and game jumps. A recent suggestion in the literature illustrates nicely aspects of material DAs [29]. The authors suggest that Lower and Middle Palaeolithic flint extraction and reduction complexes containing large limestone and knapped flint debris tailing piles that are seen from a distance could have been used as navigation markers. Notably, tailing piles are a byproduct of regular activities, that, according to this suggestion, are then noticed and used. Stable DAs may be embodied in big, stationary, objects, but they may also be embodied in mobile objects, such as tools. Populations that produce and maintain stable DAs typically have social structure, rituals and belief systems that play a role in their production, but the adaptive value is tied to the stable ecological construction itself.
3. *Dynamically stable DAs*—These cases involve distributed information that is maintained and stable through the activities of the population that produces it but is not otherwise meaningfully stable. Specifically, this category draws attention to cases in which there is a strong (possibly mathematical, dynamical) link between the properties of a population and properties of a DA. Arguably, not all cases of long-term ephemeral DA display such a strong connection, and DAs may only require a minimum population size. Examples in non-humans are: pheromone trails in ants; internal features of termite mounds. In humans perhaps the most striking case is language. Language consists primarily of transient signals, yet words do not constantly disappear simply because they have not been used in a while. Rather, words are used in various frequencies, in such a way that the population as a whole maintains these frequencies mostly stable. These stable frequencies are maintained by a diverse population of speakers, at different life stages, and with different interests and activities. Various changes to the population, in particular its size, may result in change in the size of the vocabulary or the frequencies of words. Many other factors are of course involved, and active research is being done on the relationship between population features and language features [30–32].

 A more speculative case is that of relationships between a population of artefacts coupled to a population that produces them. Taking a cue from the current crop of studies of the relationship between language and its speaker population, it would be interesting to see models of acquisition of tool-making that explore the effects of the number and types of tutors and diversity of examples that learners encounter in their learning. To what extent is exposure to a diversity of styles and artefacts at different phases of production a necessary condition for successful skill acquisition, and how does it influence later creativity of the learner?

 The study of stable, long running traditions, including traditional dance, folk stories and songs, may also benefit from considering them as dynamically stable DAs. Other phenomena that may usefully be classified in this category of DAs are traditions such as initiation ceremonies, in which social codes are embedded and information is passed; seasonal gatherings, which include implicit and explicit transfer of information of various kinds; burial ceremonies; gift exchange in the Kalahari; trade of stone tools across hundreds of kilometres in ancient times; and regional markets that attract sellers and buyers from an expansive range.

4. *Intentional DAs*—These cases involve the intentional production of an external information store (as such) and require symbolic abilities and intentional action. Examples range from cave drawings to maps and road systems (stable, intentional DAs) as well as collective decision making in humans (ephemeral, intentional DA). These technologies enable sharing and transmission of information while also, possibly as an unforeseen benefit, supporting new kinds of information and increasing the amount and stability of information a population has access to.

The evolutionary stability and hence evolutionary and ecological dynamics are potentially different in each of the four categories, as we discuss below. Roughly, the different categories differ in the speed of change and the stability of the DA, with ephemeral DAs having the shortest lifespan, and materially and dynamically stable DAs surviving longer, while it is difficult to generalize about intentional DAs. Relatedly, as we progress down the list, the origin of the DA becomes trickier. Ephemeral and materially stable DAs may arise spontaneously while dynamically stable DAs and intentional DAs reflect lager evolutionary developments. From a theoretical perspective, the categories differ in the robustness of the DAs to changes in population size, and thus to population bottlenecks.

Distinguishing between the four cases is for theoretical purposes. Whether a phenomenon is best understood as belonging to one of the four categories may require careful study. Different characteristics of a certain phenomenon might best be categorized in different categories. For example, if a technology of production of tools can be acquired by observing completed artefacts, these artefacts may constitute a stable DA. However, if it turns out that to maintain the technological industry it is necessary to have multiple copies of tools, at various stages of production, then the phenomenon would fall under dynamically stable DA; if it also requires instruction provided by another individual, which is socially organized, such as teaching, elements of it might be best described as an ephemeral DA. In fact, combining the theoretical analysis of the different kinds of DA with empirical evidence about the stability and longevity of past technologies, to support their classification to one of the four cases, may help develop further hypotheses about how they were maintained over time.

The purpose of this paper is to argue that over the course of human evolution human groups made use of various kinds of DAs, for several ecological ends; to offer general observations about several kinds of DAs and about their potential cognitive and social dimensions; and to consider whether DAs played a role in colonization of new areas and niches. We begin by presenting two examples in slightly more detail.

## 4. Detailed example 1: trail and road systems as distributed adaptations

Trail and road systems facilitate efficient and low-cost navigation at several spatial and temporal scales and in multiple ways, from easing the need to focus attention and energy on each stride and tread of a foot, to allowing navigation to places that one has never visited previously. Such systems provide crucial adaptive value to animals and humans, as movement over the landscape may often have a large influence on an individual's fitness and on a population's survival; such uses may include, for example, foraging, hunting, escaping from predators or long-distance forays for migration, raw material acquisition or trade. Trail systems evolve over time, constructed intentionally or through repeated use and wear, and develop to scales that are far beyond the ability of a single individual to construct and to even encompass in personal experience or memory. An example of such a road system is that of the Roman Empire, which developed over centuries of continuous use and elaboration and included at its peak 400 000 km of roads, more than 80 000 of them stone-paved [33]. This allowed anyone to travel anywhere in the known world with minimal need of orientation in space: one could simply follow the road, using milestones that were set along it for navigation [34]. For human societies, inter-population connectivity may be crucial for many reasons [35–37], and the possibility of relatively safe and rapid travel over long distances—even those that had not been travelled previously—constitutes an adaptation of major importance.

## 5. Detailed example 2: songs and oral traditions for navigation found among Aboriginal peoples of Australia

The cultural repertoire of modern societies is vast, and even merely its aspects that are related to technology cannot be manifested by any single individual. DAs in the form of cultural specialization into professional guilds, in the sense that knowledge is partitioned among different subgroups within society, seems to have been common in societies after the Neolithic revolution [38–45]; importantly, and although often less obvious to an external observer, there are clear examples of such distribution of knowledge also in hunter–gatherer societies, even though many of them are overall fairly generalist in the sense that most individuals can carry out most technological tasks [39,46–50]. An interesting example of distribution of crucially adaptive information developed among some groups of Aboriginal hunter–gatherers in Australia [51–53] regarding information about the lay of the land. These groups needed to travel long distances on certain rare occasions: to reach water sources during a drought; to obtain unique raw materials such as flint from distant quarries; to visit far-away related groups; or to go on pilgrimage to sacred sites. The necessary information transcended single groups’ typical range and was maintained in the form of songs, body paintings and drawn signs. In these, the features of the landscape such as paths, mountains, water holes, streams and path crossings were typically embedded within a mythical depiction of the world and the forces that act or acted in it. What is interesting from a DA perspective is that the information in some cases was assigned explicitly to retention by individuals as part of their process of coming of age, within a group/tribal framework of geographical knowledge, which in turn interacted with such frameworks of neighbouring groups whose lands lay along the routes of the rare long-distance forays that each group—or members of it—undertook occasionally [51,54].

## 6. Did distributed adaptations play a role in the dynamics of human evolution?

We hope to have convinced the reader that human evolution is replete with DAs. Some of the key notions discussed in the literature on human evolution are special cases of DAs, in particular the rather slippery notion of cumulative culture. Our aim here is to consider whether the notion of DA sheds light on transitions in human evolution and specifically to highlight connections between human social complexity and the dynamics of DAs. Put differently, what are the evolutionary implications of humans’ use of various kinds of DAs? What evolutionary possibilities or affordances were opened by them and what constraints did the reliance on DAs pose on human populations?

Lamm & Kolodny [1] identified several factors that affect DAs. Roughly, the key factors affecting the evolutionary dynamics of cultural DA discussed there are the population size and the number of individuals that need to take part in a DA in order to sustain it (parameter *A* in Lamm & Kolodny [1]). One obvious implication is that large populations are less restricted in the range of potential DAs that they might sustain, assuming that different DAs may require different values of *A*. The parameter *A* may be influenced by many factors. These include the degree to which the knowledge must be distributed in order to be maintained, for example because individuals are exposed to it randomly over a period of time. *A* may also depend on more subtle conditions, such as: the density of the population, which affects the number of interactions with potential models (i.e. denser populations have lower *A* values); the willingness of knowers to share information or to teach, e.g. degree of patience (i.e. willingness to share leads to lower *A* values); or patience of the learner. The social organization of knowledge also affects *A*: the division of the population into subgroups with different types of knowledge or whether the knowledge is secret or shared would be crucially important. Thus, both changes in the knowledge and its representation and organization, and changes in the social organization of knowledge, may affect *A* in different ways.

Each of the four categories of DA defined above has different implications for *A*. In *ephemeral DAs*, the information is embodied in the population rather than possessed by individuals and depends directly on population dynamics. Short-term ephemeral DAs are involved in group decision making and coordinated activities. Long-term ephemeral DAs such as oral traditions and rituals survive over long periods of time, without being codified and without having the entire distributed knowledge being possessed by any single individuals (consider for example songs and oral narratives). A slightly more complex form of an ephemeral DA is when division of labour is necessary because of the type of activity being performed—for example, when several things must be done simultaneously. The notion of DA becomes relevant when the role of specific features of the demography or ecological context is important for understanding how the adaptive information is maintained.

Another situation in which the ephemeral DA perspective becomes helpful is in collective decision making, in which social division of labour of the sort just described is coupled with the cognitive abilities of individuals to synthesize and combine knowledge from multiple sources (recall the vulture foraging case mentioned earlier). Long-term ephemeral DAs are particularly susceptible to changes in population size; these may be buffered in cases in which populations are partially inter-connected, as in cases of shared language or traditions across large spatial expanses. Many ephemeral DAs must rely on the abilities of individuals to coordinate and cooperate, possibly—if they encapsulate crucial adaptations—increasing the selection pressure on these abilities. They may also rely on other cultural practices to help maintain them, as when games are used to teach children group behaviours in which they must participate later in life, such as hunting and animal husbandry [55].

In *materially stable DAs*, *A* can be smaller because individuals make use of external information in the constructed environment. The adaptation may thus survive even in the face of population bottlenecks or when there are obstacles to teaching or learning. Alternatively, a materially stable DA may support division of labour, because a smaller population needs to be dedicated to the particular challenge, allowing the population as a whole to develop other kinds of knowledge.

In *dynamically stable DAs* (e.g. language) the cultural adaptation is more sensitive to properties of the population, which may in turn lead to less stability: changes in population size, degree of interactions and so on, may lead to changes in the adaptive information, up to the adaptation being lost. As in the case of ephemeral adaptations, interactions between groups may increase the cultural population size, helping to buffer against changes in any single population, but since these cases involve more fine-tuning between the population dynamics and cultural knowledge the result may be an increased rate of change (such as in the case of emergence of language dialects). Language families, consisting of multiple languages with shared features, may partly result from such considerations of population size and may even provide population size support for features that small populations are not able to maintain. A practical use stemming from this observation is that modern information technologies may allow languages spoken by small populations to survive by linking several small communities, thus increasing the relevant population size.

Since materially stable DAs are more robust to population changes, we might expect them to be more common than they seem to be in human culture. This is one puzzle prompted by the DA perspective; another puzzle is what might be called *the ephemerality of language conundrum*. Assuming that language existed for a long stretch of time prior to writing, one might ask: why was this so? Why was writing not invented earlier than it was? We of course do not propose to answer these questions here, merely to note that the DA perspective helps notice them and indicates some aspects of these puzzles that should be considered. Specifically, the decreased stability of spoken language and its greater dependence on population size might have led us to expect that material aids for its retention would develop early on. Taking this thought experiment a step forward (notably, it is nothing more than that), it would be interesting to consider the possibility that the genetic assimilation of linguistic abilities could have partly been the result of the low stability and the sensitivity to bottlenecks of ephemeral/dynamically stable DAs. These need not be restricted to ostensibly linguistic features (e.g. grammar) and may include aspects of theory of mind, joint attention, turn-taking behaviour, and motivation to communicate or coordinate. Another hypothesis that may be generated from this line of thought is that language was perhaps primarily used for coordination purposes, or for dynamic social information, rather than for storage and transmission of big amounts of knowledge or of complex knowledge. The latter would benefit from an external medium, while writing is not needed or particularly useful for real-time coordination. Again, we mention these ideas here just to illustrate considerations prompted by the notion of DAs.

Consider now several of the relatively widely agreed-upon major transitions in social complexity in human evolution. The notion of social complexity typically refers to social differentiation, technological specialization, levels of political integration and social stratification, as well as the emergence of writing and money. All are related to DAs. The latter two, writing and money, are specific DAs (for knowledge and for value). The other aspects of social complexity readily translate to the kinds of considerations illustrated in the preceding paragraphs. Agriculture and fixity of residence, with possibly related changes in population sizes and density, in particular, are relevant for considering the role of DAs in human society, given how density and population stability affects the stability of DAs, and the effect of population size on the amount of knowledge that can be ‘stored’ by distributing it in society. Specialization is a special, weak case of DA, in which information is partitioned among individuals or subgroups (so it is not a DA in the strict sense). Clearly, humanity now has a technological culture that depends on knowledge that cannot be maintained by one individual, and indeed even the renaissance men of the Renaissance did not know all that their culture knew (see in this context the insightful description of the Burke expedition in 1860 in [56]). These considerations naturally invoke further thinking about gene–culture coevolution: considering that culture in essence is a DA raises the question of the role of population size in the extent and rate of gene–culture coevolution. One potential prediction is that given DAs’ reliance on population size, gene–culture coevolution would have been most likely to occur in large populations, dense populations, or well-connected networks of populations, directing search for such dynamics to the Upper Palaeolithic and later periods. Conversely, there may be a selection pressure to genetically assimilate less stable features of DAs, so as to make individuals less sensitive to population changes that change or destroy the DA. Truly, some of the clearest examples for gene–culture coevolution relate to agricultural practices [57–60].

## 7. The conditions in which distributed adaptations would emerge or be lost and their implications

Considering human evolution from the perspective of DAs highlights affordances and constraints that may otherwise have gone underappreciated; a complementary perspective that may be of utility is a consideration of the conditions that determine the emergence and the retention of DAs, reviewed here in brief.

As discussed previously, DAs crucially rely on population dynamics and accordingly would emerge and be retained only in large-enough populations, dense-enough populations, or populations with sufficient connectivity among them. This suggests that a population that experiences significant fluctuations in these factors might lose DAs, with diverse possible outcomes: this may lead to selection in favour of decreased reliance on DAs; it may lead to selection in favour of materially stable DAs; or it may render certain potential adaptations unavailable for certain populations or in certain regions with resource or climatic fluctuations that lead to fluctuations in populations size. If successful establishment in a certain habitat required certain DAs, or if human populations grew to rely on DAs, extreme environmental conditions that set a low population carrying capacity might have prevented colonization for significant periods of time in certain regions such as the Siberian Arctic [61]. Alternatively, they might have allowed only certain human cultures and not others to establish in places whose colonization required passage through a population bottleneck: a population with minimal reliance on DAs may be better adapted to colonization of new regions [45].

This may have played a role, for example, in the early colonization of the Sahul by modern humans with a relatively simple cultural toolkit, and the surprising failure of later human establishment events in Australia until recent millenia. Human reliance on cultural DA seems to have increased significantly during these later periods, possibly limiting their ability to sustain a viable culture when passing through extreme bottlenecks such as those required for long-distance sea-faring using the period's sailing technology. Australia was first colonized by hunter–gatherers, possibly as long as 65 000 years ago [62–64]. Since then, although some evidence exists for interactions with other populations/cultures, such as arrival of domesticated dogs and sailing technology [65–67], it seems that none of these cultures succeeded in establishing there until recent millennia. Different environmental explanations have been proposed to explain what made migration to Australia possible in a specific timeframe [68,69]. The DA perspective suggests an additional factor that may have played a role: early hunter–gatherers may have been able to migrate to Australia through extreme bottlenecks without losing a substantial part of their cultural repertoire. However, if later societies that attempted to settle in Australia were reliant more heavily on DAs, for example in the form of a much larger technological toolkit and more extensive cultural knowledge to support their subsistence pattern, they would have needed to migrate in much larger numbers to be able to sustain their culture [45]. Such migration in large numbers was not feasible until the development of advanced sailing techniques in recent millennia. DAs often constitute public goods and are the product of cultural and/or physical niche construction; the conditions in which these may emerge and persist, as well as their link to cumulative culture and cultural group selection, have been the focus of much research and debate [2,5,70–81].

Studying processes leading to DAs or affected by them, like the social intelligence hypothesis and the social intelligence–ecological complexity hypothesis, is related to niche construction approaches, in that the evolution of human cognition is taken to be affected by the physical and cultural environment humans create [82]. By focusing our attention on the DA itself, DAs highlight that there are multiple ways in which this feedback can occur and that they have potentially different evolutionary preconditions and consequences. Furthermore, a population may depend on multiple DAs with different properties. Finally, DAs may depend on learning and knowledge transmission that go beyond person-to-person social learning. Taken together these observations lead to a more fine-grained analysis of particular cases of cultural niche construction. We do not attempt to summarize the findings of cultural niche construction theory but note that future study may accordingly benefit from creating a distinction between intentional and non-intentional DAs and between costly and cost-free (or incrementally beneficial) types of DAs. More speculatively, we suggest that while free-riding on DAs is possible, this may often—depending on the specific nature of the DA—have lower harmful impact on others, since a small number of cheaters will have small effect on the distributed adaptive information. Thus DAs may buffer against the evolutionary consequences of cheating.

A particular family of cases of interest are those of learning complex skills. Consider a case in which one needs to learn from multiple teachers to learn some skill properly. There may be various reasons for this. Many studies focus on learning of cultural traits from a single individual, as in learning from a parent or choosing among models according to each individual's prowess or prestige [83]. However, learning from one individual may be impossible because, for example, it is possible that no one individual produces enough examples to learn from in the time-period required for learning or because teaching beginners/intermediates/experts requires specific teaching skills, such that a single individual would not be an effective teacher during all stages of acquiring the skill. There are more extreme cases: experts often rely on tacit skills and may not know how they do what they do or recall the key steps in learning how to do it, which served as stepping stones and were later discarded. At the same time, they may be the only ones who can teach or support the final stages of learning to produce new experts. It is debatable if Mozart had within him what it took to culturally produce another Mozart (though, arguably, Bach did). Similarly, the inspiring account provided by Faulkner & Becker [84] of what it takes for musicians to make real-time improvisation music together, as in playing jazz, demonstrates that for some skills, learning from many different demonstrators is essential. At the population level, the population may not need or cannot sustain enough experts to teach enough of those starting out, not all of whom would progress to become experts, to produce the next cadre of experts. It may be more cost-effective to have many teachers for basic skills, which may even be repurposed to multiple needs. Consider the ratio of the number of mathematics teachers to professors of mathematics. How can such DAs for maintaining complex skills emerge and be sustained? Arguably, these are the most interesting and possibly also the most important ones in human DAs, those that are at the frontier of cultural development, the most extreme cases of cumulative culture—where we stand, as the saying goes, on the shoulders of giants. We leave these questions to future study but point out that many complex skills are not characterized as present or absent, offering the possibility that they could emerge gradually, with initially little reliance on an extensive cadre of tutors, and gradual development of the complexity of the skill or of the field, selecting, facilitating, or growing to rely—step by step—on an increasingly complex learning process.

One interesting corollary of this scenario relates to the debated link between population size and cultural complexity [6,35,85–92]. It suggests that such a link may exist for a reason different from the one that features in the models of cultural evolution that have figured in this discussion so far. In these models (e.g. [88,91]), cultural loss occurs owing to stochastic transmission failure, in which all learners in a certain generation fail to learn the trait successfully. Transmission is explicitly or implicitly assumed to occur between individuals, and a larger population simply increases the number of teachers and learners, decreasing the probability that all individuals would fail to learn in a coincidentally coordinated manner. Rather, the scenario highlighted here predicts that there may be cultural traits for whose retention a large number of teachers is necessary in itself.

Finally, let us point out briefly several interesting scenarios related to inter-population connectivity and DAs. (1) Some DAs may span large distances; the Australian example described earlier of encoding of spatial information in individuals’ and groups’ songs and body inscriptions incorporated many groups, across hundreds of kilometres. Such DAs may influence inter-population dynamics and be dependent on them, possibly providing incentives for peace-keeping among groups or even facilitating other modalities of interaction that ‘hitchhike’ on the DA [22]. This perspective diverges from the emphasis on cultural group selection and inter-population rivalry that is common in discussions of cumulative culture and emergent group-level traits [2,5,93]. The two perspectives are obviously not mutually exclusive. (2) Some DAs span temporal distances: material DAs such as constructed terraces for agriculture, water collection or irrigation systems, piles of mining debris or tailing discarded during stone tool production may all remain long after the population that contributed to or established them is long gone. This may facilitate rapid recolonization of previously colonized regions, for example, and may also imply that initial colonization of a region is much harder than recolonization, a readily testable prediction. Conversely, a population that relies on a materially stable DA, whose origin and design are opaque, may face difficulties if it must migrate. (3) Reliance on DAs may select in favour of increased inter-population connectivity, and in particular, may determine certain typical distance or mode of human dispersal across the landscape. Different DAs used for navigation may impose different distance limits: a rough estimate is 100 km for a single brain memory capacity, 1000 km for Aboriginals relying on songs as cognitive maps, and 10 000 and more for travellers along marked roads. If DAs are complex to an extent that they require more individuals for their maintenance than are typically found in the basic unit of spatial expansion, dispersing units (family, band, etc.) may be limited to move to short distances away from their population of origin until growing in numbers in the new location; otherwise necessary DAs may be lost.

## 8. The origins of DA

How do DAs arise? In Lamm & Kolodny [1] we argued that animal activities may spontaneously lead to useful population-level patterns that individuals may come to rely on. This can happen when individuals can recognize, even if imperfectly, and utilize collectively produced information resources. Not surprisingly, humans are able to do this, and these abilities probably go back to early hominins. Noticing the collectively produced external navigation aids such as trail systems nicely illustrates how this might happen, and how a DA may be based on humans’ behavioural plasticity and cognition. Two important properties are significant for considering the possible origin of these DAs: that they were unavoidable byproducts of necessary activities and that they are stable enough.

It is natural to compare DAs to niche construction since some of the most visible examples such as navigation cues and irrigation systems involve such changes. However, not all DAs are simply modifications of the physical environment. They intimately depend on population structure and stability (see Lamm & Kolodony [1] for further discussion). It may also be supposed that DAs are an aspect of humans’ adaptation to the cognitive niche, and that considering them separately does not further our understanding of the evolution of human cognition. However, DAs are more fine-grained, and a population may make use of several DAs, possibly of different kinds, each with its own properties and effects. Moreover, different DAs may exert conflicting pressures or affordances. For example, a DA of ecological knowledge of a relatively large area may be best supported by a relatively egalitarian social structure, which allows fission–fusion of groups and knowledge consolidation, or simply lead to it, while knowledge of hunting techniques, in that same population, might be best handled through a more hierarchical social structure.

In general terms, stable populations are better able to maintain stable DAs. They may thus become dependent on such information, which may become a source of fragility. However, as the tailing pile example illustrates, some DAs may also serve to overcome loss of adaptation to local conditions in the case of extreme population fluctuations or even of local extinctions, possibly with the support of social institutions such as rituals and belief systems which may encode efficiently, via few individuals, implicit adaptive information that has accumulated for long periods of time and/or via the trial-and-error of many individuals.

## 9. Concluding remarks

The intuition that culture is, at least in a metaphorical sense, an externalization of knowledge is widespread, and various scholars have tried to develop it in various ways [3,4,94–98]. These accounts are fruitful, but taking this analogy too far is fraught. There are two common misconceptions. The first is that externalization requires, or may even be identical to, a change in hardware: from brains or genes to physical artefacts that can carry information across time and space through symbols or language. The second misconception is conflating externalization of knowledge with making it collective, and in particular with providing group benefits. Our account of DAs moves away from the metaphorical connections between culture and memory, and by offering a well-circumscribed notion of distribution of adaptive information, it avoids the overly quick moves just mentioned. DAs are characterized functionally, based on evolutionary dynamics, and not based on the physical storage medium.

There does not exist a unified, formal framework for studying cultural adaptations as information storage systems. The literature on evolution and major transitions has addressed the notion of information systems [99]; however, it does not deal systematically with knowledge that transcends the individual. The notion of DA helps address this gap. One important advantage of this perspective is that it makes it possible to discuss different kinds of externalization and their properties prior to and independently of the use of symbolic thought or written language (cf. [95]), and is not restricted to humans. A second important advantage is that the notion of DA does not consider culture as one entity, homogeneous in organization, that evolved through a series of a small number of discrete transitions or jumps. Nor does it presuppose that changes in the organization of cultural knowledge go hand in hand with changes in cognitive abilities. Rather, DAs are fine-grained and describe the properties of particular cultural adaptations. A population may have many DAs: for navigation, for hunting, for foraging, and so on, and each may have different properties. Finally, in contrast with most other approaches, DAs make an explicit connection between cultural knowledge (in particular its amount or storage capacity), properties of the population (demography and how able the population is to maintain the DA), and ecological context.

We have emphasized cases in which DAs could have played a role in macro-cultural evolution, and in contrast with previous discussions of externalization we did not discuss the possible connection between distributed knowledge and cognition. However, one particular connection is worth highlighting since it suggests avenues for future research. This is the possible role of DAs in developmental scaffolding of human cognition [100]. A lot of suggestive examples have been discussed in the psychological literature, and integrating this evidence with formal evolutionary analysis may be instructive. Specifically, games may be DAs for social knowledge and emotion training. Many games that are played during development are ritualized traditions in various cultures, that are maintained over many generations without being symbolically codified. Psychologists have noted the connection of games to impulse control in children, to learning about rule-governed behaviours and norms, and to development of imagination and abstraction (e.g. [98,101–105]). It has been suggested that mother–child games assist the development of turn-taking behaviour and reversible role relationships between mother and child [101]; more cases are reviewed in [105]. Notice that the games themselves may be socially acquired DAs, which support the development of species-typical cognition in human children. Toys are material, external, DAs that may be scaffolds for cognitive and emotional development and, in older children, for the transmission of culturally specific social rules and roles. Given the breadth of these examples, an intriguing hypothesis is that in human evolution developmental scaffolding is the most significant use of DAs.

DAs link population size and social organization with technological practices (in Stone Age archaeology these are referred to as stone tool industry). DAs may require a population of a certain minimum size, or survive only in populations not suffering from demographic shocks. Because social or demographic changes may make a DA disappear or be less robust, they may push to new ways of distributions of the relevant information, thereby playing a role in the likelihood that a new technological DA be adopted. Alternatively, a transition in technology may play a role in population dynamics and social change. These connections may affect the possibility of reverting to older social organizations or technological systems. Quantitative models and evidence are needed to seriously assess these possibilities.

We conclude with a bold conjecture: major changes in the course of human evolution involved changes in the distribution of adaptive cultural information, in the broad sense of the word used throughout our discussion. In this paper, we have outlined some of the considerations and qualifications needed to develop this conjecture into a productive research programme and have illustrated the multiple kinds of DAs found in human culture, their multiple uses, and how DA may have affected cultural macro-evolution. We believe this thought paradigm will serve as a useful perspective in the study of major transitions in human evolution.

## Data Availability

This article has no additional data.
